# Stability of Drinking Reductions and Long-term Functioning Among Patients with Alcohol Use Disorder

**DOI:** 10.1007/s11606-020-06331-x

**Published:** 2020-11-12

**Authors:** Katie Witkiewitz, Henry R. Kranzler, Kevin A. Hallgren, Deborah S. Hasin, Arnie P. Aldridge, Gary A. Zarkin, Karl F. Mann, Stephanie S. O’Malley, Raymond F. Anton

**Affiliations:** 1 Department of Psychology, University of New Mexico, MSC 03-2220, Albuquerque, NM 87131 USA; 2 Department of Psychiatry, University of Pennsylvania, Philadelphia, PA USA; 3 Department of Psychiatry and Behavioral Sciences, University of Washington, Seattle, WA USA; 4 Department of Epidemiology, Columbia University, New York, NY USA; 5 Behavioral Health Research Division, RTI International, Research Triangle Park, NC USA; 6 Central Institute of Mental Health, Medical Faculty Mannheim, Heidelberg University, Mannheim, Germany; 7 Yale School of Medicine, New Haven, CT USA; 8 Department of Psychiatry and Behavioral Sciences, Medical University of South Carolina, Charleston, SC USA

**Keywords:** World Health Organization risk drinking levels, alcohol use disorder, reduced alcohol consumption, alcohol treatment outcomes, low-risk drinking, alcohol dependence

## Abstract

**Background:**

The World Health Organization (WHO) categorizes alcohol consumption according to grams consumed into low-, medium-, high-, and very-high-risk drinking levels (RDLs). Although abstinence has been considered the ideal outcome of alcohol treatment, reductions in WHO RDLs have been proposed as primary outcomes for alcohol use disorder (AUD) trials.

**Objective:**

The current study examines the stability of WHO RDL reductions and the association between RDL reductions and long-term functioning for up to 3 years following treatment.

**Design and Participants:**

Secondary data analysis of patients with AUD enrolled in the COMBINE Study and Project MATCH, two multi-site, randomized AUD clinical trials, who were followed for up to 3 years post-treatment (COMBINE: *n* = 694; MATCH: *n* = 806).

**Measures:**

Alcohol use was measured via calendar-based methods. We estimated all models in the total sample and among participants who did not achieve abstinence during treatment.

**Key Results:**

One-level RDL reductions were achieved by 84% of patients at the end of treatment, with 84.9% of those individuals maintaining that reduction at a 3-year follow-up. Two-level RDL reductions were achieved by 68% of patients at the end of treatment, with 77.7% of those individuals maintaining that reduction at a 3-year follow-up. One- and two-level RDL reductions at the end of treatment were associated with significantly better mental health, quality of life (including physical quality of life), and fewer drinking consequences 3 years after treatment (*p <* 0.05), as compared to no change or increased drinking.

**Conclusion:**

AUD patients can maintain WHO RDL reductions for up to 3 years after treatment. Patients who had WHO RDL reductions functioned significantly better than those who did not reduce their drinking. These findings are consistent with prior reports suggesting that drinking reductions, short of abstinence, yield meaningful improvements in patient health, well-being, and functioning.

**Supplementary Information:**

The online version contains supplementary material available at 10.1007/s11606-020-06331-x.

## INTRODUCTION

Alcohol use disorder (AUD) is highly prevalent and has high social and economic costs,^1^ yet most individuals with AUD never seek treatment.^2^ A primary reason reported by individuals with AUD for not seeking treatment is that they were not ready to stop drinking.^2^ Thus, a commitment to a goal of abstinence is an extremely high criterion for engaging individuals in treatment for AUD and determining whether alcohol treatment is successful.^3^ Drinking reductions, short of abstinence, are associated with considerable improvements in both health and functioning in general population and treatment samples.^4–9^ Broadening the definition of “success” in AUD treatment to include reduced drinking could decrease the public health burden of AUD by engaging more individuals in treatment for the disorder,^3,10^ and would provide more options for clinicians to offer patients, beyond recommending abstinence from alcohol.

### Defining “Success” in AUD Treatment

**Fig. 1:**
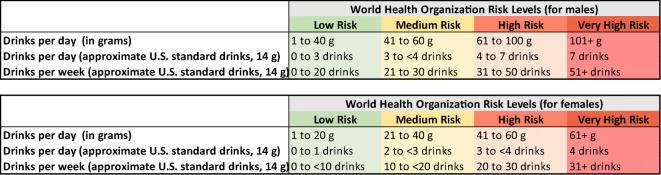
World Health Organization (WHO) risk drinking levels based on grams per day and approximate US standard drinks (14 g of pure alcohol) per day and per week. A 2-level reduction in WHO risk drinking level could include reductions from very high to medium, high to low, or low risk to abstinence. A 1-level reduction in WHO risk drinking level could include reductions from very high to high, high to medium, medium to low, or low risk abstinence.

The Food and Drug Administration^11^ recommends sustained abstinence or no heavy drinking days (defined as more than 3 drinks in a day for women and 4 drinks in a day for men) as indicators of success for AUD treatment. The European Medicines Agency^12^ (EMA) recommends abstinence, but also considers reduced drinking as a successful intermediate outcome. The EMA outcome includes reductions in total alcohol consumption, heavy drinking days, or World Health Organization (WHO) risk drinking levels (RDLs),^13^ which are defined by the number of grams (g) of alcohol consumed per day, including abstinent (0 g males/females), low risk (1 to 40 g males/1 to 20 g females), medium risk (41 to 60 g males/21 to 40 g females), high risk (61 to 100 g males/41 to 60 g females), or very high risk (101+ g males/61+ g females). The WHO RDLs provide concrete guidance for defining levels of drinking (see Fig. 1 for daily and weekly US approximate standard drink equivalents) associated with specific levels of risk and, relatedly, offer targets for reducing drinking (e.g., a two-level reduction from very high risk to medium risk). Recent studies have shown that reductions in WHO RDLs are associated with improvements in health and functioning among population samples^5,6,8^ and individuals in AUD treatment.^4,7,9^

### Current Study

Abstinence has long been considered a stable outcome for individuals with AUD,^14,15^ and numerous studies have questioned whether “controlled” drinking can be maintained.^15–17^ The debate over whether controlled drinking is achievable has been considered in the academic literature for nearly 50 years,^18^ but concrete guidance has not emerged on the magnitude of the reduction that is necessary for sustained health benefits. More recent work has shown that drinking reductions, as assessed by the WHO RDLs, are often seen over the first post-treatment year,^7^ but whether these reductions can be maintained for longer periods and whether WHO RDL reductions, short of abstinence, are associated with sustained improvements in functioning remain unclear. Given the common perception that individuals with severe AUD have difficulty sustaining reduced drinking,^3,19^ it is also unknown whether the severity of AUD at baseline moderates these effects. To address these gaps in the literature, we examined whether WHO RDL reductions of at least one level (e.g., very high to high) or at least two levels (e.g., very high to medium) can be maintained and whether these reductions in drinking are associated with long-term improvements in functioning. We hypothesized that one- and two-level reductions in WHO RDLs, even among individuals who did not achieve abstinence, would be maintained and associated with better mental health, quality of life, and functioning 3 years following treatment.

## METHODS

### Participants and Procedures

We used data from the COMBINE Study^20^ and the outpatient sample of Project MATCH,^21^ two multi-site randomized clinical trials. Participants in the current analyses met criteria for AUD, received outpatient treatment, and constituted the subgroup of participants who were followed for up to 3 years post-treatment (COMBINE: *n* = 694; MATCH: *n* = 806). Exclusion criteria for both studies included a current drug use disorder (other than nicotine or cannabis), a psychiatric disorder requiring medication, or an unstable medical condition.

#### COMBINE Study

Participants in the COMBINE Study (*n* = 1383) met criteria for alcohol dependence based on the Diagnostic and Statistical Manual of Mental Disorders (DSM) (fourth edition)^22^ and engaged in heavy drinking prior to the baseline assessment. Participants were randomized into one of nine treatment cells and received 16 weeks of treatment via a 2 × 2 × 2 design with eight of nine cells receiving (1) active naltrexone (100 mg/day) or placebo naltrexone, (2) active acamprosate (3000 mg/day) or placebo acamprosate, and (3) medication management (MM) or combined behavioral intervention (CBI) with MM. The ninth cell received CBI and no pills.

Follow-up assessments were completed at the end of treatment (16 weeks post-randomization) and at 156 weeks post-randomization. Of the 1383 participants in the COMBINE trial, 874 patients from nine sites (*n* = 1144; 76.4%) consented to a longer-term follow-up,^23,24^ of whom 694 (79%) provided data at the 3-year assessment.

#### Project MATCH

Participants in the outpatient sample of Project MATCH (*n* = 952) met the criteria for alcohol abuse (4.6%) or alcohol dependence (95.4%) based on the DSM (third edition-revised)^25^ and were actively drinking prior to the baseline assessment. Participants were randomized to receive 12 weeks of treatment with cognitive behavioral therapy,^26^ motivational enhancement therapy,^27^ or twelve-step facilitation.^28^

Follow-up assessments were completed at the end of treatment (12 weeks post-randomization) and at 156 weeks post-randomization. Of the 952 outpatients in MATCH, 806 patients (84.7%) completed the 3-year follow-up assessment.

### Measures

#### Alcohol Dependence Severity

The Alcohol Use Inventory (AUI)^29^ was administered as a measure of dependence severity in MATCH. The Alcohol Dependence Scale (ADS),^30^ which is a modified version of the AUI, was administered in COMBINE. For the current study, we included the 23 items from the ADS and the AUI that were overlapping (Supplementary Table 1) assessing alcohol dependence severity in both studies^31^ (*α* = 0.85).

#### Alcohol Consumption

Standard drinks (14 g of absolute alcohol) were measured using calendar-based methods^32,33^ to document daily drinking amounts. WHO RDLs (abstinent, low risk, medium risk, high risk, very high risk) were calculated based on the average grams of alcohol consumed per day over 1-month time periods in the month prior to the baseline assessment, in the month prior to the end of treatment, and in the month prior to the 3-year follow-up. We then calculated reductions in RDLs from baseline to the end of treatment (predictor) and from baseline to the last month of the 3-year follow-up (outcome), defined by either a one-level reduction (i.e., reducing from very high to high, high to medium, medium to low, or low to abstinent) or a two-level reduction (i.e., reducing from very high to medium, high to low, or medium to abstinent). A one-level reduction was distinguished from no change or an increase in the WHO RDL from baseline to the treatment/follow-up months. A two-level reduction was distinguished from a one-level reduction, no change, or an increase in the WHO RDL from baseline to the treatment/follow-up months. The one- and two-level reductions are not mutually exclusive categories, because anyone who achieved at least a two-level reduction also achieved a one-level reduction.

#### Functioning Outcomes at 3 Years Following Treatment

In COMBINE, mental health was assessed using the 6-item mental health subscale from the 12-item Short Form Health Survey (SF-12),^34^ with higher scores indicating better mental health functioning (*α* = 0.82). Quality of life (QoL) was assessed by a 25-item version of the WHO QOL-BREF,^35^ a measure of general QoL^36^ that covers physical, psychological, environmental, and social health domains. Higher scores indicate better QoL.

In Project MATCH, drinking consequences were assessed with the Drinker Inventory of Consequences (DrInC)^37^ and psychosocial functioning was assessed using the Psychosocial Functioning Inventory (PFI).^38^ The DrInC^37^ is a 50-item measure on which higher scores indicate more consequences (*α* = 0.97). The PFI^38^ social behavior subscale includes 10 items that assess the frequency of problematic social behavior and social interactions. Higher scores on the PFI indicate better social functioning (*α* = 0.83).

### Statistical Analysis

The primary analyses examined the maintenance of one-level and two-level WHO RDL reductions and the association between such WHO RDLs in the last month of treatment and functioning at the 3-year follow-up. To test the maintenance/stability of reductions, we examined the association between WHO RDL reductions achieved in the last month of treatment (predictor) and WHO RDL reductions achieved at the 3-year follow-up (outcomes) using logistic regression. Given both studies provided the same data for the logistic regression, we combined the data from the two studies to obtain a pooled estimate of the stability of WHO RDL reductions. We used linear regression to test the associations between WHO RDL reductions from baseline to the end of treatment (predictors) and functional outcomes at the 3-year follow-ups (outcomes). Because the two studies had different functional outcome measures at 3-year follow-ups, we conducted the linear regression analyses within each study separately. Unstandardized regression coefficients from the linear regression models can be interpreted as the differences in outcomes for patients with, versus without, one- or two-level RDL reductions, controlling for covariates. We also examined whether the severity of alcohol dependence at baseline moderated the relations between RDL reductions and functional outcomes.

All models were estimated with M*plus* version 8.2^39^ using maximum likelihood with robust estimation to account for clustering within sites.^40^ Missing drinking and functional outcome data at the end of treatment and 3-year follow-up were accommodated via multiple imputation with 50 imputed datasets.^41^ Thus, we included all individuals who completed the 3-year follow-up assessments for both studies (MATCH *n* = 806; COMBINE *n* = 694). All variables examined in the current study were included in the imputation models. Consistent with prior analyses in the COMBINE Study data,^4,9^ we controlled for the following covariates in all analyses: age, sex, race, alcohol dependence severity, smoking status, and baseline WHO RDL.

#### Non-abstinent Reductions

To evaluate the effect on the results of individuals with total abstinence, the primary analyses were repeated with abstainers excluded from the models.

## RESULTS

### Descriptive Analyses

Demographic characteristics and descriptive statistics for all measures from both studies are provided in Table 1. Most participants (90.3%) were at the medium, high, or very high RDL at baseline, so a one-level reduction was possible for all participants and a two-level reduction was possible for nearly all participants. There were no significant demographic differences and no significant differences in baseline WHO RDLs between individuals based on whether they completed the 3-year follow-up.

**Table 1:** Demographics, WHO Risk Drinking Levels at Baseline, End of Treatment, and 3-Year Follow-up, and Functional Outcomes at Baseline and 3-Year Follow-ups by Study Among 3-Year Follow-up Completers

	COMBINE (*n* = 694)			MATCH (*n* = 806)		
	Baseline	End of treatment	3-year follow-up	Baseline	End of treatment	3-year follow-up
Demographics						
Age, mean (SD)	44.9 (10.3)			38.6 (10.7)		
Male (%)	70.4%			77.2%		
Non-Hispanic White (%)	78.3%			79.4%		
Black/African American (%)	10.0%			6.0%		
Hispanic (%)	6.9%			12.0%		
American Indian/Alaska Native (%)	1.2%			1.7%		
Asian	0.1%			0.1%		
Multi-racial (%)	1.9%			0.0%		
“Other” race (%)	1.5%			0.7%		
WHO risk drinking levels						
Abstinent, *N* (%)	0 (%)	260 (37.6%)	300 (45.0%)	0 (%)	331 (41.6%)	309 (38.3%)
Low risk, *N* (%)	17 (2.4%)	270 (39.0%)	161 (24.1%)	128 (15.9%)	302 (37.9%)	267 (33.1%)
Medium risk, *N* (%)	51 (7.3%)	60 (8.7%)	63 (9.4%)	111 (13.8%)	55 (6.9%)	74 (9.2%)
High risk, *N* (%)	143 (20.6%)	45 (6.5%)	71 (10.6%)	178 (22.1%)	43 (5.4%)	67 (8.3%)
Very high risk, *N* (%)	483 (69.6%)	57 (8.2%)	72 (10.8%)	389 (48.3%)	65 (8.2%)	89 (11.0%)
At least 1-level reduction from baseline, *N* (%)		606 (87.6%)	548 (82.2%)		641 (80.5%)	602 (74.7%)
At least 2-level reduction from baseline, *N* (%)		513 (74.1%)	454 (68.1%)		498 (62.6%)	451 (56.0%)
Functional outcomes						
SF-12 mental health, mean (SD)	40.83 (11.28)	48.67 (9.78)	47.45 (10.81)			
Environmental QoL, mean (SD)	29.33 (5.55)	31.00 (5.57)	31.38 (5.29)			
Social QoL, mean (SD)	9.76 (2.64)	10.81 (2.57)	10.90 (2.57)			
Psychological QoL, mean (SD)	20.79 (4.00)	22.55 (4.26)	22.80 (4.06)			
Physical QoL, mean (SD)	27.02 (4.34)	29.02 (4.33)	28.33 (4.66)			
DrInC scores, mean (SD)				46.73 (22.23)	33.55 (25.08)	33.11 (24.89)
PFI social behavior, mean (SD)				3.22 (0.49)	3.41 (0.47)	3.43 (0.47)

All descriptive statistics are observed with no imputation for missing data. *SF-12*, Short Form Health Survey, where higher scores indicate better mental health; *QoL*, World Health Organization Quality of Life Scale, where higher scores indicate better quality of life; *DrInC*, Drinker Inventory of Consequences Scale, where higher scores indicate more alcohol-related consequences; *PFI*, Psychosocial Functioning Inventory, where higher scores indicate better psychosocial functioning

### Primary Analyses

We used logistic regression to examine the odds of maintaining one- and two-level reductions in WHO RDLs at the 3-year follow-up. In a pooled analysis of data from both studies, 83.8% and 68.0% achieved at least one-level or two-level reductions, respectively, at the end of treatment, and 84.9% and 77.7% of those participants maintained at least one-level or two-level reductions, respectively, at the 3-year follow-up. Patients with RDL reductions at the end of treatment had a significantly higher odds of achieving the RDL reduction at the 3-year follow-up for both the one-level (OR = 1.74; 95% CI: 1.59, 1.88; *p <* 0.001) and the two-level reductions (OR = 1.72; 95% CI: 1.60, 1.85; *p <* 0.001), indicating an ability to maintain/sustain the end of treatment RDL reductions at the 3-year follow-up. The logistic regression models indicated no significant interaction effects with alcohol dependence severity in predicting the maintenance of RDL reductions at the 3-year follow-up (all *p >* 0.08).

**Fig. 2:**
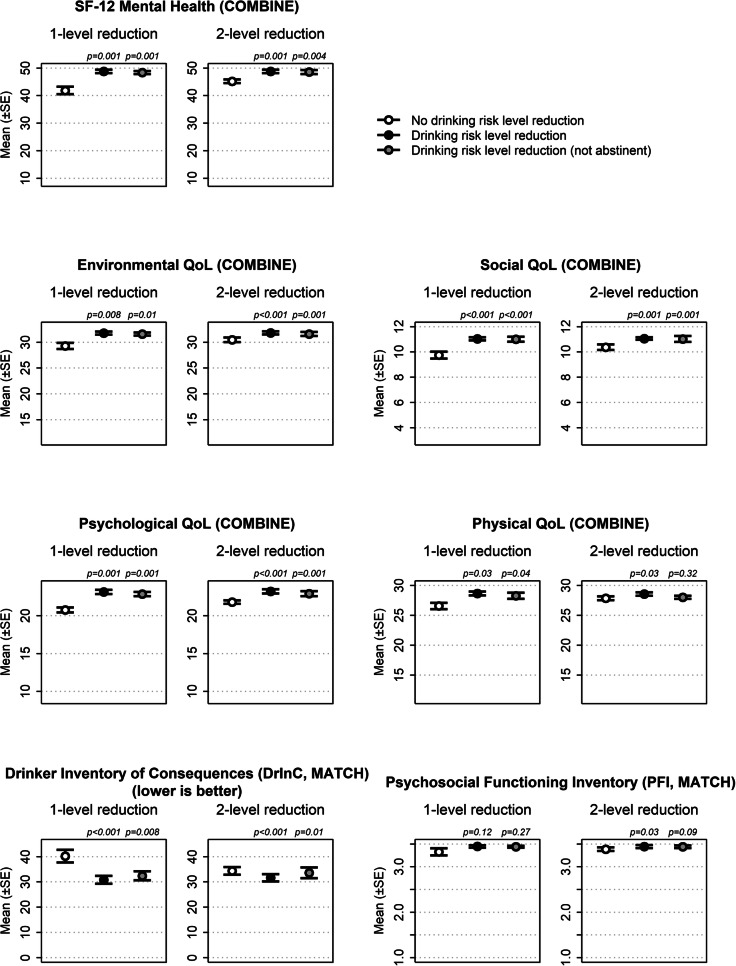
Relative difference in functional outcomes assessed at the 3-year follow-up based on risk drinking level reductions achieved at the end of initial treatment, adjusted for other covariates in linear regression analyses. Functional outcomes at the 3-year follow-up based on achieving at least a one- or two-level reduction at the end of treatment (versus achieving no change or increase in drinking risk), controlling for covariates, with *p* values from linear regression analyses comparing the one- and two-level reductions to the reference group. *Y*-axis scaling based on the lower bound for each measure. The reference group for the one-level reduction was no change or an increase in the WHO RDL from baseline to the last month of treatment. The reference group for the two-level reduction was the one-level reduction, no change, or increase in the WHO RDL from baseline to the last month of treatment. SF-12, Short Form Health Survey, where higher scores indicate better mental health; QoL, World Health Organization (WHO) QOL–BREF scale, where higher scores indicate better quality of life; DrInC, Drinker Inventory of Consequences Scale, where higher scores indicate more alcohol-related consequences; PFI, Psychosocial Functioning Inventory, where higher scores indicate better psychosocial functioning.

Next, we used linear regression to examine the association between achieving at least one- and two-level reductions in WHO RDLs at the end of treatment and functional outcomes at the 3-year follow-up in each study separately. Full results from regression analyses are provided in Supplementary Table 2. Figure 2 shows functional outcomes among those who did and did not achieve drinking reductions in the last month of treatment, adjusted for covariates. Achieving at least a one-level RDL reduction in the last month of treatment, versus no change or an increase in RDL, was associated at the 3-year follow-up with significantly better mental health; greater environmental, social, psychological, and physical QoL; and significantly fewer drinking consequences; but no difference in psychosocial functioning. Achieving at least a two-level RDL reduction in the last month of treatment, versus a one-level RDL reduction, no change, or an increase in RDL, was associated with significantly better mental health and QoL, significantly fewer drinking consequences, and significantly better psychosocial functioning.

**Fig. 3:**
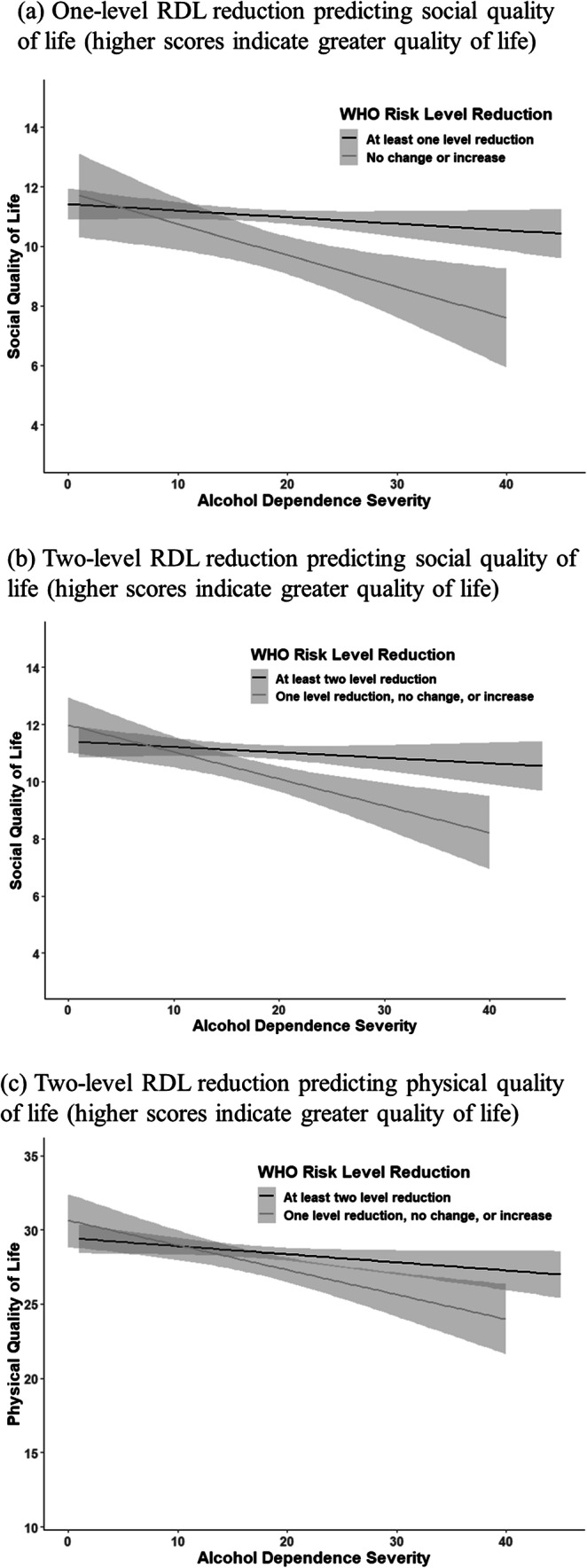
Interaction between baseline alcohol dependence severity and WHO risk drinking level reduction achieved at the end of treatment in predicting quality of life outcomes at the 3-year follow-up assessment in COMBINE. **a** One-level RDL reduction predicting social quality of life (higher scores indicate greater quality of life). **b** Two-level RDL reduction predicting social quality of life (higher scores indicate greater quality of life). **c** Two-level RDL reduction predicting physical quality of life (higher scores indicate greater quality of life). Quality of life (QoL) was assessed by the World Health Organization (WHO) QOL–BREF scale, a measure of general QoL that covers physical, psychological, environmental, and social health domains. Higher scores indicate better QoL and *Y*-axis ranges based on observed data values.

The linear regression models indicated significant interaction effects between dependence severity and one- and two-level RDL reductions in predicting social QoL and between dependence severity and two-level reductions in predicting physical QoL (Supplementary Table 2). The interaction effect showed that reductions in the WHO RDL conferred the greatest benefits in social and physical QoL at the 3-year follow-up among individuals higher in dependence severity at baseline (Fig. 3 panels a–c), perhaps because their quality of life was lower prior to treatment.

### Non-abstinent Drinking Reductions

Next, we re-estimated all models with abstainers excluded from the analysis (*N* = 432 for COMBINE and *N* = 461 for MATCH). Reductions in WHO RDLs, short of total abstinence, in the last month of treatment were significantly associated with one- and two-level reductions in WHO RDLs at the 3-year follow-up. In a pooled analysis of data from both studies, 73.1% and 54.4% of non-abstainers achieved at least one-level or two-level reductions, respectively, at the end of treatment, and 82.6% and 73.0% of those participants maintained at least one-level or two-level reductions, respectively, at the 3-year follow-up (odds of risk drinking reduction at 3 years predicted by risk drinking reduction at end of treatment = 1.70 for a one-level reduction (95% CI: 1.53, 1.87; *p <* 0.001) and 1.57 for a two-level reduction (95% CI: 1.41, 1.72; *p <* 0.001)).

Regression analyses that removed abstainers from the analyses yielded findings similar to the prior analyses (Fig. 2 and Supplementary Table 2), although the effects on functional outcomes were smaller and the effects of two-level reductions on physical QoL and psychosocial functioning were not significant with abstainers excluded. There were also significant interaction effects between dependence severity and one- and two-level RDL reductions in predicting social QoL, and between dependence severity and two-level RDL reductions in predicting mental health (Supplementary Table 2). Non-abstinent drinking reductions during the last month of treatment conferred the greatest benefits in social QoL and mental health at the 3-year follow-up for individuals highest in dependence severity (Supplementary Fig. 1 panels a–c).

## DISCUSSION

We examined whether reductions in WHO RDLs were maintained and associated with improvements in how patients feel and function, both physically and mentally, up to 3 years following treatment in two large alcohol clinical trials. The one- and two-level WHO risk reductions at the end of treatment were largely maintained and were associated with significantly better health functioning at up to 3 years following treatment. A large majority (76–87%) of individuals who achieved one- or two-level WHO risk reductions by the last month of treatment maintained those reductions at the 3-year follow-up. Moreover, individuals who achieved one- or two-level reductions in WHO RDLs by the last month of treatment had significantly better mental health and physical QoL and fewer drinking consequences at the 3-year follow-up than those who failed to achieve such reductions.

Additional analyses that excluded abstainers and tested interactions with alcohol dependence severity were largely consistent with the primary findings, with a few exceptions. Removing abstainers from the models rendered the effects of a two-level WHO RDL reduction on physical QoL and psychosocial functioning non-significant; although the size of the effects was similar. Participants with the most severe alcohol dependence had the most to gain in social and physical QoL and mental health by reducing their drinking during treatment, even short of full abstinence.

The results from the current study are consistent with recent studies showing that WHO RDL reductions are maintained for up to 1 year following treatment^7^ and significantly associated with better patient functioning.^4,5^ The current study extends these findings by providing evidence that WHO RDL reductions are maintained for up to 3 years following treatment and associated with concomitant improvements in functioning. Consistent with recent reviews on drinking reductions,^3,10,42^ the current findings indicate that among treatment seekers with AUD, reductions in drinking are achievable, maintained, and associated with long-term improvements in functioning.

The current study depended upon available data and the inclusion/exclusion criteria used in the COMBINE Study and Project MATCH. Both studies included treatment seekers with AUD who received some form of AUD treatment (psychosocial or medications) and excluded individuals with a drug use disorder or severe psychiatric and medical conditions. Whether these findings would generalize to patients with more or less severe presentations, to patients with comorbid substance use or severe psychiatric or medical disorders, or to non-treatment seekers cannot be determined from the current study. Yet, data from a large general US adult sample have shown that reductions in WHO RDLs over a 3-year period correlate with reduced odds of current alcohol dependence, drug use disorders, depression, and anxiety disorders.^5,8,43^ Future studies should recruit more diverse patient populations and extend the current analyses by examining the maintenance of WHO RDL reductions in association with more objective markers of how patients feel and function, including physical health measures, biomarkers of alcohol consumption and organ function, medical expenditures, employment data, and functioning as reported by family/friends.

Consistent with a growing body of findings from other reports^3–5,9,42–44^ and clinical guidelines,^45–47^ our results suggest that reduced drinking, as measured by the WHO RDL reduction metric, is associated with good clinical outcomes and can be sustained over time. Some clinicians might prefer suggesting at least a 1-level reduction for patients, whereas others might prefer suggesting at least a 2-level reduction. The current study provides evidence that either recommendation is likely to be sustained and associated with meaningful improvement in how patients feel and function. This information should be disseminated to inform clinical providers and the public. The information could encourage individuals with AUD who are reluctant to participate in abstinence-focused treatment to set goals with their clinical providers that are short of abstinence yet capable of providing substantial clinical benefit.

## Electronic Supplementary Material

ESM 1 (DOCX 60 kb)

## Data Availability

The original datasets for Project MATCH and COMBINE are available from the National Institute on Alcohol Abuse and Alcoholism (http://www.niaaa.nih.gov).
