# Supplementary material for: Stability of Drinking Reductions and Long-term Functioning Among Patients with Alcohol Use Disorder
Source: J Gen Intern Med. 2020 Nov 12;36(2):404–12. doi: 10.1007/s11606-020-06331-x (PMC7878601; doi:10.1007/s11606-020-06331-x)
Supplement: Supplementary file 1 — (DOCX 60 kb) [file 11606_2020_6331_MOESM1_ESM.docx]

Supplementary Figure 1. Interaction between baseline alcohol dependence severity and WHO risk drinking level reduction achieved at the end of treatment, with abstainers excluded, in predicting quality of life outcomes at the three-year follow-up assessment in COMBINE.

1. One-level reduction, short of abstinence, predicting social quality of life (higher scores indicate greater quality of life)


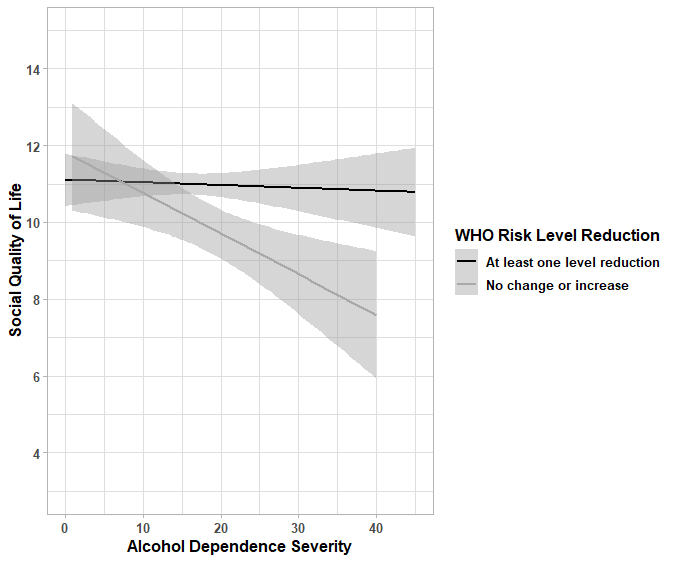


1. Two-level reduction, short of abstinence, predicting social quality of life (higher scores indicate greater quality of life)


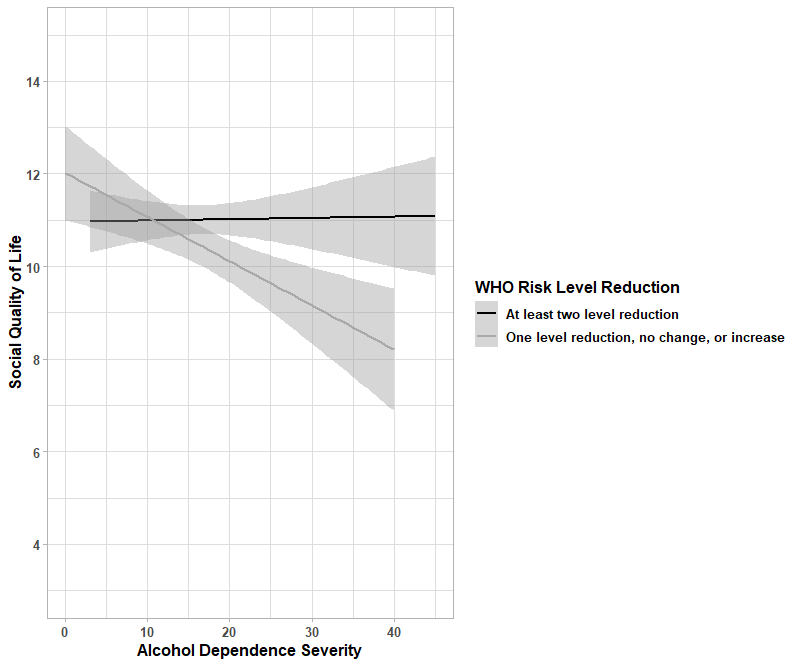


1. Two-level reduction, short of abstinence, predicting mental health (higher scores indicate greater mental health)


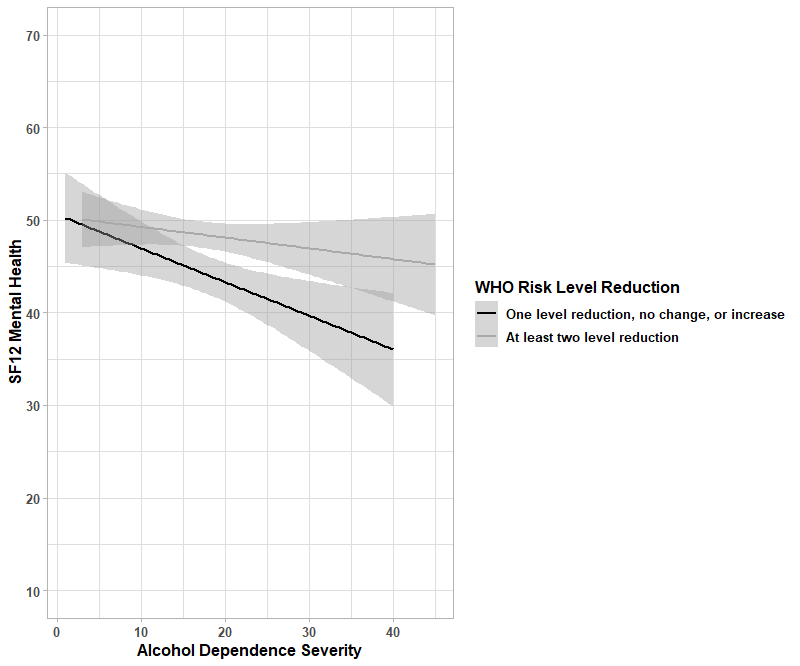


*Supplementary Figure 1 Note.* Quality of life (QoL) was assessed by the World Health Organization (WHO)QOL-BREF scale, a measure of general QoL that covers physical, psychological, environmental, and social health domains. Higher scores indicate better QoL and y-axis ranges based on observed data values.

Supplementary Table 1. *The 23 items from the Alcohol Use Inventory* ^29^ *and the Alcohol Dependence Scale* ^30^

|  |  |
| --- | --- |
| Alcohol Use Inventory ^29^ | Alcohol Dependence Scale ^30^ |
|  |  |
|  |  |
| 97. Constantly think about drinking | 18.  Constantly think about drinking/alcohol |
| 85. Gulp drinks quickly | 24.  Gulp drinks |
| 54. Drink throughout the day | 15. Drink throughout the day |
| 100. Can you stop after 1 or 2 | 25. After taking 1 or 2 drinks can you stop |
| 41. How much did you drink the last time you drank | 1.     How much drank the last time you drank |
| 44. Hangovers on Sundays or Mondays | 2.     Often have hangovers on Sunday/Monday mornings |
| 29. Have you had the shakes when sobering up | 3.     Had the shakes when sobering up |
| 59. Physically sick as a result of drinking | 4.     Got physically sick as a result of drinking |
| 87. Ever had DTs | 5.     Had the DT's (delirium tremens) |
| 71. Stumble, stagger, and weave? | 6.     Stumble about/stagger/weave when drinking |
| 88. Felt hot and sweaty | 7.     Felt overly hot and sweaty as a result of drinking |
| 28. Have you seen things not there | 8.     Seen things that weren't there as a result of drinking |
| 6. Panic if you think you will not drink when you need it | 9.     Panic when you fear you may not have a drink if need it |
| 26. Had blackouts as a result of drinking | 10.  Had blackouts (loss of memory) as a result of drinking |
| 69. Keep a bottle with you | 11.  Carry a bottle or keep one close |
| 56. Passed out from drinking | 13.  Passed out (past 12 months) as a result of drinking |
| 12. Had convulsions as a result of drinking | 14.  Had a convulsion following drinking |
| 11. Has your thinking been fuzzy or unclear because of drinking | 16.  Thinking been fuzzy/unclear after drinking heavily |
| 74. Heart beating rapidly | 17.  Felt heart beating rapidly as a result of drinking |
| 43. As a result of drinking have you heard things that aren't there | 19.  Heard things that weren't really there as a result of drinking |
| 58. Weird or frightening sensations as a result of drinking | 20.  Had weird or frightening sensations when drinking |
| 73. Felt things crawling on you | 21.  Felt things crawling on you as result of drinking |
| 106. Blackouts | 22.  Duration of blackouts (loss of memory) |
|  |  |

Supplementary Table 2. *Estimated Differences in Each Functional Outcome Assessment at up to Three Years Following Treatment as Predicted from WHO One- and Two-Level Risk Drinking Level Reductions Achieved in the Last Month of Treatment, Adjusted for Covariates in Linear Regression Models*

|  |  | |  | |  |  | |  |  |  |  |
| --- | --- | --- | --- | --- | --- | --- | --- | --- | --- | --- | --- |
|  | **COMBINE** | | | | | | | |  | **MATCH** | |
|  | SF12 Mental Health | | Environ. QOL | | Social QOL | Psychological QOL | | Physical QOL |  | DrInC | PFI |
|  | B (SE) | | B (SE) | | B (SE) | B (SE) | | B (SE) |  | B (SE) | B (SE) |
|  |  | |  | |  |  | |  |  |  |  |
| Total Sample Primary Analysis Models (COMBINE: n=694; MATCH: n=806) | | | | | | | | |  |  |  |
| 1-level reduction | 6.14 (1.89), *p*=0.001 | | 1.85 (0.70), *p*=0.008 | | 1.22 (0.33), *p*<0.001 | 2.14 (0.64), *p*=0.001 | | 1.78 (0.78), *p*=0.03 |  | -12.18 (3.48), *p*<0.001 | 0.12 (0.08), *p*=0.12 |
| 2-level reduction | 4.55 (1.41), *p*=0.001 | | 1.77 (0.43), *p*<0.001 | | 0.95 (0.28), *p*=0.001 | 1.79 (0.40), *p*<0.001 | | 1.17 (1.53), *p*=0.03 |  | -9.62 (2.66), *p*<0.001 | 0.09 (0.04), *p*=0.03 |
|  | |  | |  | | |  | |  |  |  |
| Total Sample Interaction with Dependence Severity (COMBINE: n=694; MATCH: n=806) | | | | | | | | |  |  |  |
| 1-level x severity | 0.21 (0.15), *p=*0.16 | | 0.02 (0.06), *p=*0.71 | | 0.08 (0.03), *p=*0.01 | 0.07 (0.05), *p=*0.22 | | 0.05 (0.05), *p=*0.38 |  | -0.10 (0.50), *p=*0.85 | 0.01 (0.01), *p=*0.50 |
| 2-level x severity | 0.21 (0.11), *p*=0.054 | | 0.07 (0.05), *p*=0.19 | | 0.06 (0.03), *p=*0.03 | 0.07 (0.04), *p=*0.09 | | 0.08 (0.04), *p=*0.04 |  | -0.16 (0.35), *p=*0.64 | 0.004 (0.004), *p=*0.30 |
|  | |  | |  | | |  | |  |  |  |
| Abstainers Excluded from the Primary Analysis Models (COMBINE: n=432; MATCH: n=465) | | | | | | | | |  |  |  |
| 1-level reduction | 5.91 (1.83), *p*=0.001 | | 1.64 (0.65), *p*=0.01 | | 1.20 (0.28), *p*<0.001 | 1.90 (0.58), *p*=0.001 | | 1.36 (0.65), *p*=0.04 |  | -9.22 (3.46), *p*=0.008 | 0.10 (0.10), *p*=0.27 |
| 2-level reduction | 4.67 (1.62), *p*=0.004 | | 1.50 (0.45), *p*=0.001 | | 0.95 (0.28), *p*=0.001 | 1.56 (0.46), *p*=0.001 | | 0.51 (0.52), *p*=0.32 |  | -6.73 (2.67), *p=*0.01 | 0.09 (0.05), *p*=0.09 |
|  |  | |  | |  |  | |  |  |  |  |
| Abstainers Excluded from the Interaction with Dependence Severity (COMBINE: n=432; MATCH: n=465) | | | | | | | | | | | |
| 1-level x severity | 0.28 (0.18), *p=*0.11 | | 0.04 (0.07), *p=*0.57 | | 0.10 (0.04), *p=*0.02 | 0.08 (0.07), *p=*0.21 | | 0.05 (0.07), *p=*0.46 |  | -0.003 (0.53), *p=*0.99 | 0.004 (0.007), *p=*0.56 |
| 2-level x severity | 0.26 (0.11), *p*=0.02 | | 0.08 (0.06), *p*=0.14 | | 0.09 (0.03), *p=*0.004 | 0.09 (0.04), *p=*0.05 | | 0.10 (0.05), *p=*0.06 |  | 0.09 (0.45), *p=*0.84 | 0.004 (0.007), *p=*0.57 |
|  |  | |  | |  |  | |  |  |  |  |

*Note.* B (SE)=Unstandardized regression coefficients (standard error), which can be interpreted as the change in each outcome based on achieving at least a 1- or 2-level reduction, versus no change or increase in risk drinking, at the average of all covariates (covariate effects reported in Supplementary Table 3); For example, a 1-level reduction corresponds to an average increase in 6.14 in SF-12 mental health scores at the three year follow-up, which is a considerable increase in SF12 scores given the standard deviation of 10 (thus, individuals who achieve a 1-level reduction report, on average, a nearly 2/3 standard deviation increase in mental health). SF12=Short Form Health Survey, where higher scores indicate better mental health; QOL=World Health Organization Quality of Life Scale-BREF, where higher scores indicate better quality of life; DrInC=Drinker Inventory of Consequences Scale, where higher scores indicate more alcohol-related consequences; PFI=Psychosocial Functioning Inventory, where higher scores indicate better psychosocial functioning.. The reference group for the one-level reduction was no change or an increase in the WHO risk drinking level from baseline to the last month of treatment/last month of follow-up, and the reference group for the two-level reduction was the one-level reduction, no change, or increase in the WHO risk level from baseline to the last month of treatment/last month of follow-up.

Supplementary Table 3. *Full Results for Regression Model Results for Functioning Outcomes up to Three Years Post-Treatment as Predicted from WHO One- and Two--Level Reductions at the End of Treatment*

|  |  |  |  |  |  |  |  |  |
| --- | --- | --- | --- | --- | --- | --- | --- | --- |
|  | **COMBINE** | | | | |  | **MATCH** | |
|  | SF12 Mental Health | Environmental QOL | Social QOL | Psychological QOL | Physical QOL |  | DrInC | PFI |
|  | B (SE) | B (SE) | B (SE) | B (SE) | B (SE) |  | B (SE) | B (SE) |
|  |  |  |  |  |  |  |  |  |
| Baseline WHO | -0.78 (0.40)* | -0.32 (0.24) | -0.21 (0.11) | -0.23 (0.11) | -0.54 (0.21)** |  | 1.74 (1.07) | -0.02 (0.02) |
| Age | 0.10 (0.04)** | 0.06 (0.03)* | 0.01 (0.01) | -0.003 (0.01) | -0.04 (0.02)* |  | 0.07 (0.08) | 0.01 (0.001)*** |
| Sex (male=1) | 1.54 (0.72)* | -0.79 (0.44) | -0.46 (0.19)* | 0.45 (0.23)* | -0.11 (0.28) |  | 2.81 (3.74) | 0.04 (0.04) |
| Non-Hispanic White=1 | 0.49 (1.23) | 1.95 (0.76)* | -0.29 (0.32) | 0.13 (0.55) | 1.13 (0.62) |  | -0.82 (2.11) | -0.05 (0.04) |
| ADS baseline | -0.14 (0.06)* | -0.14 (0.03)*** | -0.03 (0.01)* | -0.08 (0.02)*** | -0.08 (0.02)** |  | 0.46 (0.17)** | -0.01 (0.005)* |
| Smoker | 0.18 (1.27) | -0.96 (0.53) | -0.01 (0.19) | -0.02 (0.58) | -0.66 (0.52) |  | 3.58 (1.94) | 0.03 (0.02) |
| One-level reduction | 6.14 (1.89)** | 1.85 (0.70)** | 1.22 (0.33)*** | 2.14 (0.64)** | 1.77 (0.78)* |  | -12.18 (3.48)*** | 0.12 (0.08) |
|  |  |  |  |  |  |  |  |  |
|  | **COMBINE** | | | | | | **MATCH** | |
|  | SF12 Mental Health | Environmental QOL | Social QOL | Psychological QOL | Physical QOL |  | DrInC | PFI |
|  | B (SE) | B (SE) | B (SE) | B (SE) | B (SE) |  | B (SE) | B (SE) |
|  |  |  |  |  |  |  |  |  |
| Baseline WHO | -1.44 (0.46)** | -0.60 (0.23)* | -0.35 (0.12)** | -0.50 (0.14)** | -0.70 (0.23)** |  | 2.67 (1.11)* | -0.03 (0.02) |
| Age | 0.10 (0.04)** | 0.06 (0.02)* | 0.01 (0.01) | -0.003 (0.01) | -0.04 (0.02)* |  | 0.06 (0.08) | 0.01 (0.001)*** |
| Sex (male=1) | 1.37 (0.66)* | -0.88 (0.44)8 | -0.49 (0.19)** | 0.37 (0.22) | -0.14 (0.30) |  | 3.37 (3.80) | 0.03 (0.05) |
| Non-Hispanic White=1 | 0.73 (1.23) | 2.01 (0.77)* | -0.25 (0.33) | 0.21 (0.56) | 1.20 (0.61) |  | -0.81 (1.99) | -0.05 (0.04) |
| ADS baseline | -0.15 (0.06)** | -0.14 (0.02)*** | -0.03 (0.01)** | -0.08 (0.02)*** | -0.08 (0.02)*** |  | 0.48 (0.17)** | -0.01 (0.005)* |
| Smoker | 0.17 (1.32) | -0.94 (0.52) | -0.01 (0.19) | -0.01 (0.58) | -0.67 (0.53) |  | 3.44 (1.88) | 0.03 (0.02) |
| Two-level reduction | 4.55 (1.41)** | 1.77 (0.43)*** | 0.95 (0.28)** | 1.79 (0.40)*** | 1.17 (0.53)* |  | -9.62 (2.66)*** | 0.09 (0.04)* |

*Note. * p*<0.05; *** p*<0.01; **** p*<0.001, SF12=Short Form Health Survey; QOL=World Health Organization Quality of Life Scale; DrInC=Drinker Inventory of Consequences Scale; PFI=Psychosocial Functioning Inventory. The reference group for the one-level reduction was no change or an increase in the WHO risk drinking level from baseline to the last month of treatment/last month of follow-up, and the reference group for the two-level reduction was the one-level reduction, no change, or increase in the WHO RDL from baseline to the last month of treatment.
